# Cord blood T cell subpopulations and associations with maternal cadmium and arsenic exposures

**DOI:** 10.1371/journal.pone.0179606

**Published:** 2017-06-29

**Authors:** Unni C. Nygaard, Zhigang Li, Thomas Palys, Brian Jackson, Melanie Subbiah, Meena Malipatlolla, Vanitha Sampath, Holden Maecker, Margaret R. Karagas, Kari C. Nadeau

**Affiliations:** 1 Sean N. Parker Center for Allergy and Asthma Research, Stanford University, Division of Pulmonary and Critical Care, Division of Immunology, Allergy, and Rheumatology, Departments of Medicine and Pediatrics, Stanford University School of Medicine, Stanford, California, United States of America; 2 Department of Toxicology and Risk Assessment, Norwegian Institute of Public Health, Oslo, Norway; 3 Children’s Environmental Health and Disease Prevention Research Center at Dartmouth, Lebanon, New Hampshire, United States of America; 4 Institute for Immunity, Transplantation, and Infection, Stanford University School of Medicine, Stanford, California, United States of America; Stony Brook University, Graduate Program in Public Health, UNITED STATES

## Abstract

**Background:**

Arsenic and cadmium are environmental pollutants, and although the evidence for adverse immune effects after prenatal arsenic and cadmium exposures is increasing, little is known about the underlying immunological mechanisms.

**Methods:**

We investigated the relationship between prenatal arsenic and cadmium exposures and a variety of T cell subpopulations measured in cord blood for 63 participants in the New Hampshire Birth Cohort Study. Post-partum toenail concentrations of arsenic and cadmium were used as an estimate of maternal exposure during pregnancy. The characteristics of cord blood proportions of T lymphocytes and subpopulations (expression of markers for Th1, Th2, Th17, Th1Th17, induced and natural regulatory T cells and NKTs) are presented.

**Results:**

In regression analyses, maternal arsenic exposure levels were inversely associated with cord blood T helper memory cells (-21%, 95% CI: -36%, -3%) and the association was found to be stronger in females. They were also inversely associated with activated T helper memory cells, particularly in males (-26%, 95% CI: -43%, -3%). Similarly, inverse associations were observed between cadmium exposure levels and activated T helper memory cells (-16%, 95% CI: -30%, -1%) and also for T helper memory cells in females (-20%, 95% CI: -35%, -3%).

**Conclusion:**

The results suggest that prenatal exposures to relatively low levels of arsenic and cadmium may contribute to altered distribution of T cell populations at birth. These changes in theory, could have contributed to the previously reported immunosuppressive effects observed later in infancy/childhood.

## Introduction

Inorganic arsenic and cadmium are ranked at first and seventh, respectively, by the Agency of Toxic Substances and Disease Registry in the 2015 priority list of hazardous substances in the United States [1]. Worldwide, millions are chronically exposed via drinking water to arsenic levels above the limit of 0.01 mg/L set by the World Health Organization [2]. Further exposure can occur via the diet [3]. Food and tobacco smoking are the primary sources of non-occupational cadmium exposure, although water and air levels of cadmium may be high and contribute to cadmium exposure near industries that utilize cadmium [4].

Although the evidence for adverse immune effects after prenatal arsenic and cadmium exposures is increasing, data from human studies are limited and little is known about the underlying immunological mechanisms of these developmental contaminants. Increasing cadmium body burden in children has been reported to be associated with immunosuppressive effects [5]. Mice chronically exposed to cadmium had impaired resistance to *Listeria monocytogenes* [6]. Cadmium exposure in mothers was associated with altered DNA methylation profiles in leukocytes obtained from mothers and cord blood [7]. Overall, studies indicate the intricate effects of cadmium on innate cells like monocytes/macrophages, NK cell function, and thymus and splenic cells resulting in alterations in the number, maturation, and function of T cells [8].

Immunosuppression by prenatal arsenic exposure has been reported in a number of studies, including enhanced susceptibility to infections [3], which depends on well-functioning T cells. The observed inverse associations between exposure and infection resistance have been supported by mechanistic and animal studies [9–12]. Although most of these studies were conducted in populations exposed to high levels of arsenic, our group has recently reported increased susceptibility to infections in those exposed to chronic levels of low to intermediate exposure levels of arsenic [13, 14]. Our previous study, using data obtained from the New Hampshire Birth Cohort Study (NHBCS), suggested that maternal urinary arsenic concentration during pregnancy were associated with alterations in T cell populations in cord blood samples [15]. The focus of the present study was to investigate the relation between both prenatal arsenic and cadmium exposures on T cell subpopulations measured in cord blood, in an additional subset of the NHBCS cohort, and to expand the current state of knowledge of cord blood immunology in humans by applying state of the art methods to immunophenotyping.

## Materials and methods

### Ethics statement

The study protocols for the NHBCS were approved by the Committee for the Protection of Human Subjects at Dartmouth College. All study participants provided written informed consent.

### Study population

To be eligible for the NHBCS, women were: a) currently pregnant, b) 18 to 45 years old, c) receiving routine prenatal care at one of the study clinics, d) living at residence served by a private water system (e.g., serving <15 households or 25 individuals), e) residing in the same place since their last menstrual period, using the same water supply, and f) not planning to move prior to delivery.

### Cord blood sample collection and measurements

The study provided hospital delivery rooms with cord blood collection kits and a list of enrolled women with their expected delivery dates. Cord blood was collected from study participants at the time of delivery by hospital delivery room staff by dripping the cord blood directly into collection tubes after the cord was cut. From each participant one 8 ml BD Vacutainer^™^ Glass Mononuclear Cell Preparation Tube (CPT) (Becton Dickinson; BD 362761) was obtained. After collection, blood was delivered to the hospital laboratory, placed in a refrigerator within 2 hours of collection, and stored at 4°C until processed. When collected at remote delivery rooms, cord blood samples were transferred to processing laboratories within 24 hours via professional courier service in a cooler with frozen cooler packs. Cord blood samples were processed within 24 hours of collection as described.

CPT tubes were warmed to room temperature and inverted 8 times prior to centrifugation. Tubes were centrifuged in a Sorvall Legend RT centrifuge for 30 minutes at 1692g (2800 rpm). After centrifugation, the plasma layer above the lymphocytes was transferred in 2ml polypropylene screw cap cryovials using a transfer pipette. The lymphocyte layer was transferred by pipette to a 50 ml conical centrifuge tube, diluted up to 25 ml with PBS and centrifuged for 15 minutes at 311g (1200 rpm) to pellet the cells. After the supernatant was decanted, cells were suspended in 10 ml of PBS and centrifuged for 15 minutes at 311g (1200 rpm) to pellet the cells again. Lymphocytes were suspended in 1.5 ml of PBS, diluted 1:2 by the addition of 1.5 ml of freeze media and aliquoted as 1ml volumes into 1.8 ml Nunc cryovials. Final vial concentration of freeze media constituents was 50% FBS and 10% DMSO. Vials were placed into chilled “Mr Frosty” freezer containers and kept at -80°C for 24 hours, thereafter transferred to liquid nitrogen storage.

### Exposure assessment of postpartum toenail arsenic and cadmium levels

At two weeks post-partum, participants received an information packet requesting maternal toenail clippings within eight weeks of birth, a timing which was consistent with other studies [16, 17]. In the laboratory, maternal toenails underwent an additional washing procedure that included manual removal of visible dirt and five washes in an ultrasonic bath using Triton X-100 (LabChem Inc., PA) and acetone followed by deionized water, and allowed to dry. All toenail samples were subject to low-pressure microwave digestion and were analyzed via ICP-MS. Average within batch percent recovery of Cd from standard reference materials, spiked digested blanks and spiked samples was 94.9%, 100.5% and 97.9%, respectively. Among batches the coefficient of variation of the average percent recovery of Cd from standard reference materials, spiked digested blanks and spiked samples was 6.0%, 5.5% and 6.0%, respectively. The detection limit differs on a sample-by-sample basis because of the difference in sample weights used for digestion and analysis. The detection limit of arsenic and cadmium in maternal toenails ranged from 0.001–0.41 μg/g and 0.002–0.196 μg/g, respectively.

### Immune cell phenotyping in cord blood

Peripheral blood mononuclear cell (PBMC) samples from cord blood for 63 participants were shipped on dry ice overnight to the Human Immune Monitoring Center at Stanford University, and stored in liquid nitrogen until analyses.

The samples were thawed in smaller batches according to established methods [18]. In short, the sample were quickly thawed in a 37°C water bath and then transferred to a vial to which warm RPMI with 10% FBS and 0.001% benzonase dropwise were added. After two washes, 1 x 10^6^ cells/100μL were stained with an antibody cocktail for detection of T cell subsets: FITC -Live/dead stain, PE anti-CXCR3 (clone 1C6/CXCR3), PE-CF594 anti-CD294 (clone BM16), PerCP-Cy5.5 anti-CD4 (clone SK3), PE-Cy7 anti-CCR6 (clone 11A9), APC anti-CD127 (clone HIL-7R-M21), AF700 anti- CD3 (clone UCHT1), APC-H7 anti-CD45RO (clone UCHL1), BV421 anti-CD25 (clone 2A3), V500 anti-CXCR5 (clone RF8B2), BV605 anti-HLA-DR (clone G46-6) and BV711 anti-CD56 (clone NCAM16.2), all antibodies from BD bioscience (CA, USA). All tubes were incubated on ice in the dark for 20 min, and subsequent to two washes and resuspension in 0.3 ml staining buffer, analyzed on a LSRII flow cytometer (BD Biosciences, CA). The cell surface markers were used to identify the following subtypes of T cells: naïve (CD45RO-), memory (CD45RO+), natural T regulatory cells (CD45RO- CD127- CD25+), induced Tregs (CD45RO+ CD127- CD25+), and natural killer T cells (CD56+) in CD3+ CD4+ T cells. Additionally, markers suggestive of Th cell differentiation included CXCR3+ CCR6- for Th1, CD294+ for Th2, CXCR3- CCR6+ for Th17, CXCR3+ CCR6+ for Th1Th17. Activated cells were identified by the presence of cell surface marker HLADR+ [19]. Manual gating was performed using FlowJo version 10 software (TreeStar, Ashland, OR), and the cell subpopulations are expressed as percent of parent population.

### Statistical methods

Descriptive statistics including mean, standard deviation, median, interquartile range (IQR) and range were presented for individual T cell subsets and other covariates. Nonparametric spearman correlations among the T cell subsets were calculated and tested with p values. NK and NKT cells were not included in subsequent analyses due to the high number of missing values for these markers. A multivariate exploratory factor analysis [20] was performed using the immune cell populations (see Results section) for all individuals, in order to understand the pattern of the T cells data. Further, the K-means clustering approach was used to partition the samples based on the T cell data, and clusters of subjects were discovered using elbow criterion [21]. The K means cluster were plotted against the first factors, that explained the most variance. Nonparametric Kruskal-Wallis rank sum test for continuous variables and Chi-square tests for binary or categorical variables were used to examine the associations between the clusters and the cohort characteristics data.

To study the associations of the arsenic and cadmium exposures with T cell populations, regression analyses were performed for the estimation and testing. The exposure data and cell population variables were log-transformed to comply with normal distributions. To select potential confounders to be included in the model, we calculated the correlations between the metals and the candidate covariates including maternal age, parity, gestational age and birth weight. Covariates with Pearson correlations above 0.2 were selected. For the two binary variables—ever smoked during pregnancy and infant sex—we applied the two-sample t test to compare the metal levels and those with covariates with a marginal p-value T test result (p<0.1) were included in the model. We then tested the association of the covariates with the outcome using linear regression. As none of the covariates were related to the metal exposures and the outcomes, they were not included in our ultimate models.

We examined possible interaction effects between arsenic and cadmium, using median cut point, and including the product of the two binary variables in the regression equation.

## Results

### Study population

For this study, we selected 83 women enrolled between February 28, 2012 and April 15, 2013 on whom we collected a urine sample at study entry, a cord blood sample at delivery, and a stool sample from their infant at 6 weeks of age for subsequent microbiome analysis. Overall participation rate in this period was 76% (a total of 292 out of 384 eligible). A total of 63 samples had sufficient cell numbers for determination of the cell subpopulations. The population characteristics for the present study are shown in Table 1, including maternal age and BMI at pregnancy, smoking during pregnancy (self-reported), infant sex, gestational age and weight at birth. Maternal post-partum toenail concentrations of arsenic and cadmium were both right skewed, with geometric means of 0.05 (SD = 1.9) and 0.005 (SD = 2.5) respectively (Table 2).

**Table 1:** Selected characteristics of the New Hampshire Birth Cohort Study participants included in the present study (n = 63*).

Selected characteristics	Number (%)
Maternal age (years)	
22–29	19 (34%)
30–34	23 (40%)
35–43	15 (26%)
Parity	
0	22 (39%)
1	28 (49%)
2–4	7 (12%)
Pre-pregnancy maternal BMI	
Normal	36 (63%)
Overweight	13 (23%)
Obese	8 (14%)
Gestational Age (weeks)	
30.40–39.05	19 (33%)
39.06–39.89	18 (32%)
39.90–42.10	20 (35%)
Birth weight (grams)	
2270–3227	19 (33%)
3228–3604	18 (32%)
3605–4565	20 (35%)
Infant Sex—male	34 (60%)
Maternal smoking—yes	4 (7%)

BMI, body mass index

* There were 6 subjects missing all the above characteristic information

**Table 2:** Maternal postpartum toenail arsenic and cadmium exposure levels in the study (n = 63*).

Exposure variable	Mean (SD)	Median (IQR)	Min–Max	5% and 95% percentiles
**As in toenail clippings (μg/g)**	0.06 (0.05) GM: 0.05 (1.9)	0.05 (0.04)	0.01–0.32	0.02–0.15
**Cd in toenail clippings (μg/g)**	0.008 (0.01) GM: 0.005 (2.5)	0.004 (0.007)	0.001–0.07	0.001–0.02

GM, geometric mean

* There were 6 subjects missing As and Cd

### Cord blood T cell phenotypes

The distributions of the various T cell subsets in the PBMC samples isolated from cord blood are shown in Table 3. The majority of the T cells were naïve T helper cells (69.3%). The median proportion of activated Th cells were 0.8% and 4.4% for naïve and memory Th cells, respectively, and 3.5% for memory Th cells. The proportion of Th cells expressing markers associated with Th1 and Th17 were significantly higher than for Th2, Th1+Th17+, and natural and induced regulatory T cells. The proportion of follicular T cells (CD3+CD4+CXCR5+) in cord blood were in general low (<1%). The percentages of several T cell subsets were correlated, as reported in Table 4. The strongest association were between Th cells and the naïve Th cells (r = 0.97), and Th memory cells was significantly, positively correlated with activated naïve Th cells (r = 0.40) and with Th cells expressing markers for Th1 (r = 0.46), Th17 (r = 0.46), Th1Th17 (r = 0.43), nTregs (0.34) and iTregs (r = 0.56). The Th2 marker was not correlated with any of the other subpopulations, with the exception of the Th1Th17 population.

**Table 3:** Relative amounts of selected immune cell populations in cord blood samples collected at birth (n = 63*).

% of parent	Population markers	Mean (SD)	Median (IQR)	Min—Max
**Naive CD4+ T cells**^a^	CD3+ CD4+ CD45RO-	64.5 (20.7)	69.3 (21)	9.2–94.9
**Activated naive CD4+ T cells**^b^	CD3+ CD4+ CD45RO- HLADR+	1.9 (3.3)	0.8 (2)	0.1–19.7
**Memory CD4+ T cells**^a^	CD3+ CD4+ CD45RO+	4.4 (3.8)	3.5 (3.3)	0.4–18.9
**Activated memory CD4+ T cells**^c^	CD3+ CD4+ CD45RO+ HLADR+	6.6 (7.7)	4.4 (4.5)	0.0–48.0
**Th1+ marker**^d^	CD3+ CD4+ CXCR3+ CCR6-	5.2 (2.7)	4.6 (3.3)	0.6–14.2
**Th2+ marker**^d^	CD3+ CD4+ CD294+	1.1 (0.8)	0.9 (0.66)	0.2–3.9
**Th17+ marker**^d^	CD3+ CD4+ CXCR3- CCR6+	3.9 (1.9)	3.7 (2.3)	0.6–9.8
**Th1+ Th17+marker**^d^	CD3+ CD4+ CXCR3+ CCR6+	1.3 (1.5)	0.7 (1.4)	0.0–7.7
**nTreg**^d^	CD3+ CD4+ CD45RO- CD127- CD25+	1.2 (0.8)	1.0 (0.7)	0.0–3.6
**iTreg**^d^	CD3+ CD4+ CD45RO+ CD127- CD25+	1.1 (1.2)	0.6 (0.9)	0.1–5.6
**NKT cells**^a^	CD3+ CD56+	1.6 (1.9)	0.7 (1.7)	0.1–8.2
**Activated NKT cells**^e^	CD3+ CD56+ HLADR+	17.2 (14.4)	12.8 (20)	0.0–53.1

* Activated naive CD4+ T cells and Activated memory CD4+ T cells had two missing values. NKT cells and Activated NKT cells had 25 missing values.

All populations were gated on live cells (gate set based on the live/dead cell strain), thereafter gated on lymphocytes as determined by cell size/granularity (i.e. gate set based on FSC/SSC). The populations are expressed as % of the parent populations:

^a^CD3+,

^b^CD3+ CD4+ CD45RO-,

^c^CD3+ CD4+ CD45RO+,

^d^CD3+ CD4+,

^e^CD3+ CD56+.

iTreg, induced Treg; nTreg, natural Treg; NKT, natural killer T;

**Table 4:** Correlation matrix for the different T cell subpopulations in cord blood, spearman correlation coefficients and p-values are presented, correlations with p<0.05 denoted in bold.

	**Th**	**Th naïve**	**Act. Th naïve**	**Th mem**	**Act. Th mem**	**Th1**	**Th2**	**Th17**	**Th1Th17**	**nTreg**	**iTreg**
**Th**	1.000	**0.97, <0.001**	**-0.47, <0.001**	0.07, 0.611	-0.20, 0.133	**-0.33, 0.009**	-0.24, 0.054	0.13, 0.307	**-0.43, <0.001**	-0.21, 0.094	**-0.44, <0.001**
**Th naïve**	**0.97,** 0**<0.001**	1.000	**-0.53, <0.001**	-0.09, 0.501	-0.24, 0.064	**-0.43, <0.001**	-0.23, 0.073	0.05, 0.689	**-0.52, <0.001**	-0.24, 0.057	-**0.54, <0.001**
**Act Th naïve**	**-0.47, <0.001**	**-0.53, <0.001**	1.000	**0.40, 0.001**	**0.69, <0.001**	0.24, 0.062	0.02, 0.888	0.08, 0.562	0.22, 0.090	**0.34, 0.008**	**0.55, <0.001**
**Th mem**	0.07, 0.611	-0.09, 0.501	**0.40, 0.001**	1.000	**0.32, 0.012**	**0.46, <0.001**	-0.16, 0.224	**0.46, <0.001**	**0.43, <0.001**	**0.34, 0.006**	**0.56, <0.001**
**Act Th mem**	-0.20, 0.133	-0.24, 0.064	**0.69, <0.001**	**0.32, 0.012**	1.000	0.21, 0.109	-0.13, 0.332	0.07, 0.596	0.15, 0.263	0.14, 0.284	**0.49, <0.001**
**Th1**	**-0.33, 0.009**	**-0.43, <0.001**	0.24, 0.062	**0.46, <0.001**	0.21, 0.109	1.000	0.17, 0.173	**0.39, 0.002**	**0.65, <0.001**	0.18, 0.149	**0.50, <0.001**
**Th2**	-0.24, 0.054	-0.23, 0.073	0.02, 0.888	-0.16, 0.224	-0.13, 0.332	0.17, 0.173	1.000	0.10, 0.454	**0.42, <0.001**	-0.08, 0.539	0.12, 0.368
**Th17**	0.13, 0.307	0.05, 0.689	0.08, 0.562	**0.46, <0.001**	0.07, 0.596	**0.39, 0.002**	0.10, 0.454	1.000	**0.49, <0.001**	**0.27, 0.036**	0.16, 0.203
**Th1Th17**	**-0.43, <0.001**	**-0.52, <0.001**	0.22, 0.090	**0.43, <0.001**	0.15, 0.263	**0.65, <0.001**	**0.42, <0.001**	**0.49, <0.001**	1.000	0.17, 0.180	**0.44, <0.001**
**nTreg**	-0.21, 0.094	-0.24, 0.057	**0.34, 0.008**	**0.34, 0.006**	0.14, 0.284	0.18, 0.149	-0.08, 0.539	**0.27, 0.036**	0.17, 0.180	1.000	**0.38, 0.002**
**iTreg**	**-0.44, <0.001**	**-0.54, <0.001**	**0.55, <0.001**	**0.56, <0.001**	**0.49, <0.001**	**0.50, <0.001**	0.12, 0.368	0.16, 0.203	**0.44, <0.001**	**0.38, 0.002**	1.000

### Cord blood T cell phenotyping–Factor and cluster analysis

**Fig 1:**
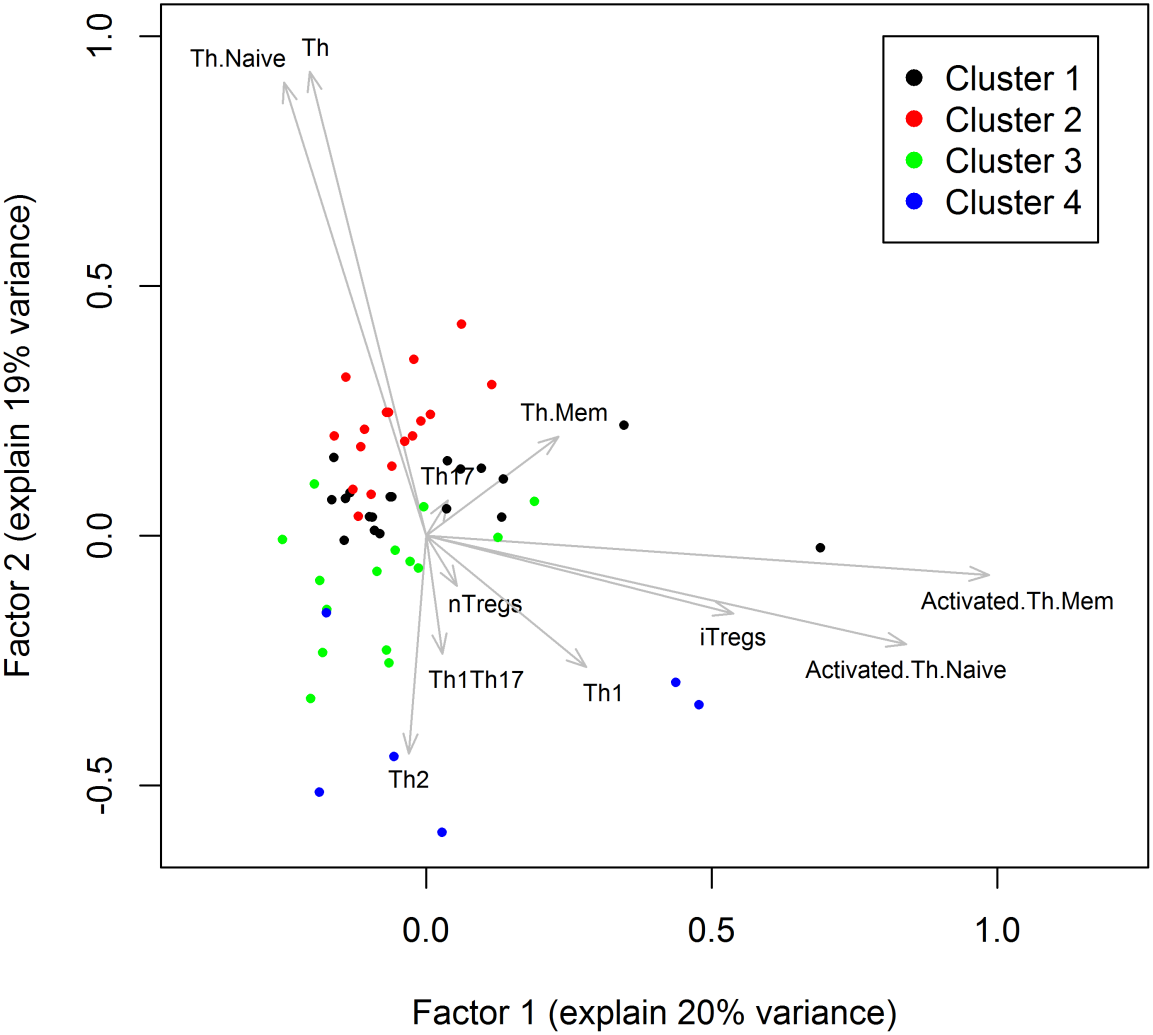
K-means clustering in relation to factors. Each individual (dot) is displayed related to factor 1 and 2, based on factor analyses of the immune cell phenotype data set for the 63 individuals. The color codes represent the 4 clusters from the K-means analyses.

The exploratory factor analysis revealed that six factors explained 80% of the total variance in the T cell phenotyping data. The first and second factors explained 20% and 19% variances (Fig 1) and the others explaining 11%, 11%, 10% and 8% respectively. The factor loadings indicated that there are three dominating variables on the first factor: activated memory Th cells (loading (l) = 0.99), activated naïve Th cells (l = 0.84) and iTregs (l = 0.54). The second factor is dominated by Th cells (l = 0.93) and naïve Th cells (l = 0.91). Thus, the main characteristics contributing to the spread of the individuals based on T cell phenotypes were the proportion of activated Th cells, i.e., the expression of HLA-DR on activated naïve and memory Th cells.

The K-means clustering approach with elbow criterion identified 4 clusters of with 19, 17, 15 and 9 individuals, respectively. These four clusters explained 82.4% of the total sum of squares suggesting a good partition. Hierarchical clustering (results not shown) gave similar clustering as the K-means clustering. Th and naïve Th cells appeared to be important drivers for the four clusters as their patterns were distinctly different across the four clusters. This is consistent with data shown in Fig 1 where the clusters assignments were superimposed on the plot of the first two factors of the factor analysis. The cluster memberships of the subjects more clearly separated along the vertical (second factor) than the horizontal direction (first factor). Individuals with high values along the second factor, dominated by Th and naïve Th cells, were more likely to be in cluster 2, and low values, dominated by Th2 cells, were more likely to be in cluster 4.

### Relationship between T cell subsets and participant characterizations

To investigate whether the immune cell subset profiles were related to characteristics like maternal age, maternal smoking, parity, birth weight, gender, maternal BMI, gestational age, postpartum toenail arsenic and postpartum toenail cadmium, we compared these variables across the four K-means clusters identified above. No apparent associations between the characteristic variables and the immune cell clusters were seen.

### Relationship between maternal arsenic and cadmium exposures and the cord blood T cell subsets

Arsenic exposure levels were inversely associated with T helper memory cells (-21% when doubling exposure level, 95% CI: -36%, -3%), and the association was stronger among females (-28%, 95% CI: -48%, 1%) than males (-17%, 95% CI: -37%, 9%) (Table 5). Arsenic levels were inversely associated with activated T helper memory cells (-22%, 95% CI: -40%, 1%); Table 5) and was statistically significant in males (-26%, 95% CI: -43%, -3%).

**Table 5:** Associations^1^ between maternal arsenic concentrations in postpartum toenail and immune cell subpopulations (n = 57*). Statistically significant associations are denoted in bold font. The beta values are the percent of changes in the relative immune cell numbers per doubling of arsenic exposure.

	Arsenic		Arsenic, female		Arsenic, male	
Immune Cell Subset	beta	95% CI	beta	95% CI	beta	95% CI
**Th**	-1%	(-12%,11%)	-7%	(-25%,15%)	3%	(-10%,17%)
**naïve Th**	1%	(-10%,13%)	-5%	(-24%,19%)	4%	(-9%,19%)
**Activated naive Th**	-14%	(-40%,22%)	5%	(-52%,129%)	-20%	(-46%,20%)
**Memory Th**	**-21%**	**(-36%,-3%)**	-28%	(-48%,1%)	-17%	(-37%,9%)
**Activated memory Th**	-22%	(-40%,1%)	-11%	(-53%,67%)	**-26%**	**(-43%,-3%)**
**Th1**	-2%	(-17%,16%)	-15%	(-40%,20%)	8%	(-8%,26%)
**Th2**	13%	(-6%,38%)	-2%	(-26%,29%)	25%	(-5%,65%)
**Th17**	-11%	(-24%,5%)	-22%	(-42%,4%)	-3%	(-19%,17%)
**Th1Th17**	9%	(-22%,53%)	4%	(-49%,111%)	10%	(-26%,63%)
**nTregs**	12%	(-9%,39%)	33%	(-11%,99%)	5%	(-20%,37%)
**iTregs**	-6%	(-29%,24%)	11%	(-30%,77%)	-15%	(-41%,21%)

^1^ The covariates maternal age, parity, gestational age, birth weight, ever smoke and sex were considered, but were not found to be confounders and thus the table shows the results from the final, univariate model.

* Sample sizes in the regression models were 57 for most cell types due to 6 subjects missing arsenic exposure and the sample size for Activated naive Th and Activated memory Th were further reduce to 55 due to two subjects missing relative amounts measurement of the two cell subtypes.

Similarly, inverse associations were observed between cadmium exposure levels and activated T helper memory cells (-16%, 95% CI: -30%, -1%), as well as with T helper memory cells, particularly in females (-20%, 95% CI: -35%, -3%); Table 6). None of the other cell subpopulations were clearly related to arsenic or cadmium exposure levels.

**Table 6:** Associations^1^ between maternal cadmium concentrations in postpartum toenail and immune cell subpopulations (n = 57*). Statistically significant associations are denoted in bold font. The beta values are the percent of changes in the relative immune cell numbers per doubling of cadmium exposure.

	Cadmium		Cadmium, female		Cadmium, male	
Immune cell subset	beta	95% CI	beta	95% CI	beta	95% CI
**Th**	3%	(-5%,11%)	-2%	(-14%,13%)	8%	(-3%,19%)
**naïve Th**	4%	(-4%,13%)	0%	(-13%,15%)	8%	(-2%,20%)
**Activated naive Th**	-15%	(-33%,7%)	-23%	(-47%,11%)	-4%	(-30%,33%)
**Memory Th**	-12%	(-24%,2%)	**-20%**	**(-35%,-3%)**	-4%	(-23%,20%)
**Activated memory Th**	**-16%**	**(-30%,-1%)**	-21%	(-41%,6%)	-11%	(-29%,11%)
**Th1**	-5%	(-15%,7%)	-6%	(-25%,16%)	-4%	(-15%,10%)
**Th2**	4%	(-9%,20%)	-3%	(-18%,15%)	12%	(-10%,41%)
**Th17**	-6%	(-16%,6%)	-10%	(-25%,9%)	-3%	(-16%,12%)
**Th1Th17**	-7%	(-26%,16%)	-10%	(-37%,27%)	-6%	(-32%,28%)
**nTregs**	8%	(-7%,24%)	-2%	(-21%,21%)	18%	(-4%,45%)
**iTregs**	-9%	(-25%,11%)	-15%	(-36%,13%)	-1%	(-26%,31%)

^1^ The covariates maternal age, parity, gestational age, birth weight, ever smoke and sex were considered, but were not found to be confounders and thus the table shows the results from the final, univariate model.

* Sample sizes in the regression models were 57 for most cell types due to 6 subjects missing cadmium exposure and the sample size for Activated naive Th and Activated memory Th were further reduce to 55 due to two subjects missing relative amounts measurement of the two cell subtypes.

To study the effects of potential outliers, we performed a sensitivity analysis by removing 5 potential outliers who had the lowest Th cell values in the data. Results from the sensitivity analysis showed same pattern for the clustering and associations of interest.

To test the interaction effects of arsenic and cadmium on T cells, we constructed a binary variable for each exposure using a median cutpoint, and then included the binary variables and their product term in the regression model. In this model, the interaction term was statistical significant for Th1Th17 (-70%, 95% CI: -91%, -13%), and of borderline statistical significance for Th1 (-43%, 95% CI: -67%, 1%) and Th17 (-42%, 95% CI: -67%, 2%).

## Discussion

Both arsenic and cadmium have been shown to exert complex effects on immune cells. In earlier studies, we reported that in utero exposure to low or intermediate levels of arsenic exposure in our pregnancy cohort was associated with a higher risk of infection in infants during the first year of life [13, 14]. In an earlier study using a different subset of participants from the NHBCS, we found that maternal urinary arsenic concentrations were inversely associated with naïve activated Th cells and positively associated with CD45RA+ CD69- CD294+ naïve non-activated Th2 cells in cord blood, indicating that chronic exposure to low levels of arsenic could alter fetal T cell populations [15]. In this study, we further assessed a number of T cell sub-populations and their activation state in cord blood from the NHBCS. Toenail clippings were used to determine concentrations of both arsenic and cadmium as their slow growth rate represents a window of exposure of about 6–12 months prior to collection (2–8 weeks post-partum) and reflect maternal exposure levels around the first trimester [22]. Our results indicate that arsenic and cadmium exposure levels are inversely associated with T helper memory cells. We did not find interaction effect of the two metals on T helper memory cells. However, common modes of action of the two metals are possible, as both metals have been reported to target the thymus, to demonstrate suppressed T cell function, and involve inflammation and NRF2 pathways [8, 23–26]. A decrease in Th memory cells in humans has been shown to increase susceptibility to infections [27]. Thus, we speculate that the impaired proportion of memory T cells at birth could be a contributing factor to the early postnatal period (first 4 months) being a particularly vulnerable period with regard to increased risk of infections [14].

We found that associations with cadmium and arsenic and T helper memory cells differed by gender, with strongest associations in females for both arsenic and cadmium. An in vitro model suggests that female cord blood cells, in particular, are more sensitive than those of males to arsenic-induced telomerase stimulation at sub micro-molar concentrations (possibly due to the increased expression of ras and myc oncogenes) [28]. In mice, exposure to environmentally relevant doses of cadmium during gestation has been reported to affect thymocyte development of the newborn (< 12 h old) offspring [29], as well as to bring about persistent changes to T cell phenotypes and immune responses to a streptococcal vaccine, which were to some extent sex-specific [8]. Neonatal cadmium exposure in rats through maternal milk showed decreased proliferation of lymphocytes in female rats, but not male rats [30]. It was further shown that cadmium interacts with 17β-estradiol and this interaction may account for the increased risk for cadmium-induced immunomodulation in females than males [31].

As expected, the proportion of activated Th cells (0.8% and 4.4% for naïve and memory Th cells, respectively) and memory Th cells (3.5%) were low compared to adult populations [32]. The strong association between Th cells and the naïve Th cells (r = 0.97) is not surprising due to the high percentage of naïve Th cells within the Th cell population. Interestingly, the proportion of Th memory cells was significantly, positively correlated with activated naïve Th cells and with Th cells with markers for Th1, Th17, Th1Th17, nTregs and iTregs, in agreement with the notion that activated/differentiated T cells will be accompanied with development of memory cells [33, 34]. Presence of memory Th cells have been reported in other studies on cord blood [35] reporting that about 20% of the total Th population were expressing memory markers (or 8% of the mononuclear cells), while Thome and co-workers [36] reported a presence of 15 and 5% of Th cells in infants to express markers for T central and effector memory cells, respectively. Whether the “memory cells” (CD3+ CD4+ CD45RO+) in fetal cord blood are of maternal or fetal origin in the present study, however, is unknown. In humans at birth, circulating T cells are in general functionally immature [37]. On the other hand, it has been shown that maternal transfer of T cells occurs [38]. Thus, potential transfer of maternal Th memory cells to the fetus should be considered. It is likely that the arsenic exposures have also affected the maternal pool of T cells, since arsenic-related immune effects (increased infections) are seen for both mother and child [39]. The putative impact on immunosuppression by the observed impairment of Th memory cells early in life is, however, irrespective of maternal or fetal origin of the cells.

Our study has certain limitations. Our sample size is relatively small; however, the power of our analysis shows trends for differences in the immune system, despite relatively low levels of exposure to arsenic and cadmium. Toenail analysis can be susceptible to external contamination, however we minimized this possibility through carefully washing.

## Conclusion

The results suggest that prenatal exposures to relatively low levels of arsenic and cadmium may contribute to altered distribution of T cell subpopulations, specifically memory cells and activated memory cells, at birth and offers a potential mechanism by which these contaminants may increase risk of infections later in childhood.

## Supporting information

S1 File Raw data. (CSV)

## Abbreviations

CPT: Cell Preparation Tube
NHBCS: New Hampshire Birth Cohort Study
PBMC: Peripheral blood mononuclear cell
